# Complete Genome Sequences of Spondweni Viruses Isolated between 1958 and 1960

**DOI:** 10.1128/MRA.01278-18

**Published:** 2018-12-13

**Authors:** Petrus Jansen van Vuren, Joe Kgaladi, Venessa Patharoo, Janusz T. Paweska

**Affiliations:** a Centre for Emerging Zoonotic and Parasitic Diseases, National Institute for Communicable Diseases, National Health Laboratory Service, Sandringham, South Africa; University of Maryland School of Medicine

## Abstract

Here, we report the complete genome sequences of 14 Spondweni viruses isolated in South Africa and Mozambique between 1958 and 1960. The sequences comprise 13 mosquito isolates and 1 human isolate following a documented laboratory infection. This study expands the publicly available data for this neglected virus from 4 to 18 sequences.

## Abstract (precis)

Here, we report the complete genome sequences of 14 Spondweni viruses isolated in South Africa and Mozambique between 1958 and 1960. The sequences comprise 13 mosquito isolates and 1 human isolate following a documented laboratory infection.

## ANNOUNCEMENT

The Spondweni serogroup in the Flavivirus genus and Flaviviridae family comprises the human pathogens Zika virus and Spondweni virus (SPOV) (1–5). Zika virus (ZIKV) was first isolated in Uganda in 1947 but was acknowledged as a pathogen with severe impacts on human health only when recent outbreaks highlighted its propensity to cause severe neurological complications (6, 7). ZIKV is sexually transmissible, and SPOV was detected in testes and semen at a low level in a mouse model (8–10).

Six human infections with SPOV have been documented since its discovery in 1952 in Nigeria (1–5). ZIKV and SPOV infections result in similar disease presentations, ranging from asymptomatic to a mild or moderate febrile illness (11). The initial misidentification of SPOV as a strain of ZIKV and the extensive serological cross-reaction between these viruses have likely resulted in the misinterpretation of a number of early serosurveillance studies in Africa (11).

Until recently, SPOV was confined to sub-Saharan Africa. However, a SPOV isolate from Culex quinquefasciatus mosquitoes in Haiti in 2016 confirms that its distribution has expanded well beyond Africa (12). SPOV was isolated from four species of culicine mosquitoes in South Africa and Mozambique between 1958 and 1960 (4). We determined the full-genome sequence of these very early isolates.

SPOV isolates initially obtained from suckling mice (4) (Table 1) were cultured in VeroE6 cells, and viral RNA was extracted from the clarified supernatants (Qiagen viral RNA minikit). Virus cDNA was prepared as described before (13). Sequencing libraries were prepared using the Nextera DNA library preparation kit as recommended by the manufacturer (Illumina, USA) and sequenced on the MiSeq Illumina platform. Random hexamer and adapter sequences were removed from the reads using Cutadapt v1.21 (14). Quality filtering was performed using Prinseq-lite v0.20.4 (15). Sequencing reads were aligned to a reference sequence (Spondweni virus SM6-V1; GenBank accession number DQ859064) to determine consensus SPOV genomes. Reads were aligned to the reference sequence using Bowtie 2 (16), duplicates were removed using Picard, and a new consensus was generated using custom scripts (https://github.com/jtladner/Scripts/tree/master/reference-based_assembly). The mean read length for the 14 samples ranged between 264 and 272 nucleotides (nt), and total reads ranged between 1,250,200 and 1,832,112 nt. The number of reads mapped to the reference per sample ranged between 1,935 and 18,483 nt. Annotation was done manually using the CLC Genomics Workbench v10.0.1. Pairwise distance calculations between Spondweni isolate sequences were performed using MEGA v7.0 software (17).

**TABLE 1:** Spondweni virus isolates sequenced in this study

Isolate no.^*a*^	Donor	Yr	Locality	Passage history^*c*^	GenBank accession no.^*b*^
AR3061	Aedes fowleri/Aedes fryeri	1960	Lumbo, Mozambique	MB 1 Vero 2	MH829612
AR2239	Aedes circumluteolus	1959	Ndumu, KwaZulu Natal, South Africa	MB 3 Vero 2	MH829611
AR2238	Aedes circumluteolus	1959	Ndumu, KwaZulu Natal, South Africa	MB 2 Vero 2	MH829610
AR2203	Mansonia africana	1959	Ndumu, KwaZulu Natal, South Africa	MB 3 Vero 2	MH829609
AR2164	Aedes circumluteolus	1959	Ndumu, KwaZulu Natal, South Africa	MB 2 Vero 2	MH829608
AR1266	Aedes circumluteolus	1958	Ndumu, KwaZulu Natal, South Africa	MB 2 Vero 2	MH829607
H127	Homo sapiens	1958	Laboratory infection	MB 2 Vero 2	MH829613
AR1168	Mansonia africana	1958	Ndumu, KwaZulu Natal, South Africa	MB 3 Vero 2	MH829606
AR1163	Mansonia africana	1958	Ndumu, KwaZulu Natal, South Africa	MB 2 Vero 2	MH829605
AR1086	Aedes circumluteolus	1958	Ndumu, KwaZulu Natal, South Africa	MB 3 Vero 2	MH829604
AR1084	Mansonia africana	1958	Ndumu, KwaZulu Natal, South Africa	MB 1 Vero 2	MH829603
AR1081	Eretmapodites silvestris	1958	Ndumu, KwaZulu Natal, South Africa	MB 3 Vero 2	MH829602
AR1077	Aedes circumluteolus	1958	Ndumu, KwaZulu Natal, South Africa	MB 2 Vero 2	MH829601
AR1071	Aedes circumluteolus	1958	Ndumu, KwaZulu Natal, South Africa	MB 1 Vero 2	MH829600

a All genomes are 10,290 bases in length and have a GC content of 52.5%.

b Consensus sequences after reference mapping are available in the public database GenBank with accession numbers given above. Raw sequencing reads, in the form of FASTQ files, were deposited in the Sequence Read Archive (SRA) under BioProject accession number PRJNA501801.

c MB, suckling mouse brain.

As expected, the 14 SPOV isolates were closely related to previously sequenced SPOV isolates. The overall pairwise nucleotide distance between SPOV isolates was 0.61% (range, 0% to 2.31%). The nucleotide sequences of isolates AR1084 and AR1086 were identical, as were the sequences of AR1077 and AR1081 (Table 1). The sequence of AR3061 was most closely related to previously sequenced isolates, with a pairwise nucleotide distance ranging from 0.31% to 2.25%. The sequence of the recent Haiti isolate was the most divergent but was still closely related to all other isolates, with a pairwise nucleotide distance between 0.76% and 2.31%. The sequence similarities between AR1168 and H127 suggest that the laboratory infection (4) of the donor of H127 occurred while working with isolate AR1168.

The low level of divergence of the 2016 Haiti isolate from older isolates is surprising, considering the 50-year lapse between isolations, suggesting that the Spondweni virus genome is relatively conserved. Our sequence data expand the publicly available sequences for SPOV to 18. These additional historical isolate sequence data provide a more comprehensive baseline of sequences for future phylogenetic analyses and should enable the development of more accurate diagnostic tools.

### Data availability.

Final sequences were deposited in GenBank and raw reads in the SRA. The accession numbers are provided in Table 1.
